# Enrichment of a subset of Neanderthal polymorphisms in autistic probands and siblings

**DOI:** 10.1038/s41380-024-02593-7

**Published:** 2024-05-17

**Authors:** Rini Pauly, Layla Johnson, F. Alex Feltus, Emily L. Casanova

**Affiliations:** 1 Biomedical Data Science and Informatics Program, Clemson University, Clemson, SC 29634 USA; 2 Department of Psychology, Loyola University, New Orleans, New Orleans, LA 70118 USA; 3 Department of Genetics and Biochemistry, Clemson University, Clemson, SC 29634 USA; 4 Center for Human Genetics, Clemson University, Clemson, SC 29634 USA

**Keywords:** Autism spectrum disorders, Genetics

## Abstract

*Homo sapiens* and Neanderthals underwent hybridization during the Middle/Upper Paleolithic age, culminating in retention of small amounts of Neanderthal-derived DNA in the modern human genome. In the current study, we address the potential roles Neanderthal single nucleotide polymorphisms (SNP) may be playing in autism susceptibility in samples of black non-Hispanic, white Hispanic, and white non-Hispanic people using data from the Simons Foundation Powering Autism Research (SPARK), Genotype-Tissue Expression (GTEx), and 1000 Genomes (1000G) databases. We have discovered that rare variants are significantly enriched in autistic probands compared to race-matched controls. In addition, we have identified 25 rare and common SNPs that are significantly enriched in autism on different ethnic backgrounds, some of which show significant clinical associations. We have also identified other SNPs that share more specific genotype-phenotype correlations but which are not necessarily enriched in autism and yet may nevertheless play roles in comorbid phenotype expression (e.g., intellectual disability, epilepsy, and language regression). These results strongly suggest Neanderthal-derived DNA is playing a significant role in autism susceptibility across major populations in the United States.

## Background

DNA evidence taken from human remains from the Middle Pleistocene indicate that anatomically modern humans (AMH) and other archaic humans underwent multiple introgression events [1]. Of those archaic humans, *Homo neanderthalensis* (Neanderthals) has received the most attention, providing the most fossil material and occupying the position as our closest known cousin on the hominin tree of life. It has been estimated that Eurasian-derived populations have approximately 2% Neanderthal DNA, which was acquired during introgression events occurring shortly after AMH migrated out of Africa [2, 3]. These hybridization events occurred somewhere between 47–65 thousand years ago (kya) [4]. A subset of Europeans later immigrated back into Africa approximately 20 kya, bringing some of this Neanderthal ancestry with them, such that all modern Africans have a small but measurable amount of Neanderthal DNA from the event [5].

With the recent sequencing of multiple archaic human genomes, there has been growing interest concerning the influence of archaic human-derived alleles on modern health [6, 7]. With regards to Neanderthal-derived variants, previous groups have identified positive selection on genes relating to immune function, skin and hair pigmentation, physiological responses to high altitude conditions, aspects of metabolism, hypercoagulation, and propensity for depression [6, 8].

In general, dosage-sensitive genes are tightly conserved and resistant to such introgression events [9, 10]. Most genes involved in brain development follow this dosage-sensitive pattern and have tended to be resistant to introgression [11]. In support of this, Srinivasan et al. [12] found a depletion of Neanderthal-derived variants within autism- and other brain-related genes in the general population. On the other hand, other studies have reported a number of non-synonymous and other single nucleotide variants within neural genes that have been implicated in the condition [3, 13].

Additional research involving non-clinical populations has identified strong links between certain brain and skull morphologies and enrichment of Neanderthal DNA [14, 15]. Specifically, enrichment is associated with reduced globularity in the skull shape of modern populations, a finding mildly reminiscent of the elongated skull morphology characteristic of Neanderthal and other archaic crania [15]. Enrichment of Neanderthal DNA is also associated with enhanced neural connectivity within visual processing systems, particularly between the intraparietal sulcus (IPS) and the occipital cortex and fusiform gyrus, and decreased connectivity within the default mode (social) network [14, 16].

Importantly, many of these same connectivity patterns are recapitulated in autism, which is a major impetus for the current work. For instance, underconnectivity within the default mode network is consistently reported in autism [17]. Meanwhile, increased connectivity within visual processing networks is also a commonly reported feature. Keehn et al. [18] tested autistic children^1^ using a visual search task in which this group tends to excel. They identified an “island of sparing” in autism reflected in increased connectivity within occipital regions and enhanced connectivity of these visual processing areas to the frontal lobes. Autistic people also often have cognitive strengths in areas such as mathematics. Iuculano et al. [20] found that during numerical problem solving, autistic children had greater activation in the fusiform gyrus and occipital lobes compared to non-autistic children, again suggesting that visual processing modalities are an area of strength in autism. Interestingly, many of these features are shared by non-clinical groups with high Neanderthal DNA content [14, 15].

In light of this evidence, in the current study we addressed whether Neanderthal DNA is enriched in autistic people and their siblings compared to ethnically-matched controls. We accessed whole exome sequencing (WES) for autistic probands and unaffected siblings from the Simons Foundation Powering Autism Research (SPARK) Database [21] for comparison against individuals in the Genotype-Tissue Expression (GTEx) and 1000 Genomes (1000G) databases [22, 23]. Significant enrichment in the autism group was especially driven by rare Neanderthal-derived variants, but also some common variants, which suggests weak but ongoing purifying selection towards removal of some of these single nucleotide polymorphisms (SNP) from the human genome.

## Materials and methods

An assemblage of Neanderthal-derived SNPs was provided by the Sankararaman laboratory and has been previously published [7, 24]. Only European-specific SNPs were used in the current study due to European admixture across these various ethnic groups. The authors applied for and received access to WES from the SPARK dataset (Clemson University IRB2018-235), which is a collection of extensive genotype and clinical data on autistic individuals and their unaffected siblings [21]. Samples have been collected from a wide range of collection sites within the US and can be found on the SFARI website (see: https://www.sfari.org/resource/spark/). Only individuals of self-reported “white” (white Hispanic, white non-Hispanic) and “black” (black non-Hispanic) backgrounds were used in these analyses; any participant listed as “admixed” was removed. Autistic individuals with potential confounding genetic and environmental factors were also excluded from the study. These conditions include: congenital anomalies, birth or pregnancy complications, premature birth, fetal alcohol syndrome, and cognitive delays due to exposure or medical condition. Ethnically matched controls were accessed from the GTEx Project (Clemson University IRB2022-0589) for comparison with the black non-Hispanic and white non-Hispanic SPARK groups respectively. Unfortunately, GTEx includes too small of a sample (<2%) of Hispanic individuals for adequate comparison, so these were removed from the GTEx sets and a different control group was used for white Hispanics in the SPARK group [25]. For white Hispanics, the Mexican (MXL) and Puerto Rican (PUR) ancestry subgroups from 1000G were used as controls— data that are freely available for download [23]. According to the Pew Research Center, people of Mexican ancestry currently compose about 60% of all Hispanics within the mainland US, while Puerto Ricans compose another 9% [26]. Together, these two groups of Hispanics compose the largest subset of Hispanic people in the mainland US and are available in 1000G, and for this reason these subgroups have been selected to approximate a control group, utilizing a 1:6 (PUR : MXL) ratio in order to best mimic the Pew data.

Allelic frequencies were determined using ethnically matched control groups. While numbers of white non-Hispanic exomes were sufficient (GTEx N = 706, SPARK affected = 2004, SPARK unaffected siblings = 172), the number of black non-Hispanic SPARK affected and GTEx individuals were comparatively small (SPARK N = 72, GTEx N = 102), which limited our analysis of this latter subset (see Table 1 for basic demographics). In addition, the white Hispanic control group was similarly limited (N = 74), which has likely affected the Rare NeanderScore results, creating the appearance of an even greater divergence than probably exists; therefore, these results should be interpreted with some caution. All SNPs with missing data were removed from the analyses due to significant fluctuations in results when even small amounts of missing data were retained (e.g., even a 5% threshold for missing data dramatically altered results with the current study design). After removal of 546 SNPs with missing data, this left a total of 1288 SNPs for comparison across all groups.

**Table 1:** Basic demographics of participants.

Ethnic group	Group	Total number (N)	Males	Females
Black, Non-Hispanic (BNH)	Control	102	70	32
	Autism	72	61	11
White Hispanic (WHS)	Control	74	40	34
	Autism	312	255	57
White, Non-Hispanic (WNH)	Control	706	465	241
	Autism	2004	1567	437
	Siblings	172	142	30

To process the genotype calls from SPARK, GTEx, and 1000G VCFs, the individual genotypes were converted into a binary call format (bcf) using bcftools (see: https://github.com/samtools/bcftools). Subsequently, the bcf files were transformed into genotype matrices using the JVARKIT tools developed by Pierre Lindenbaum. The JVARKIT tools can be found at the following GitHub repository (see: https://github.com/lindenb/jvarkit/).

**Fig. 1:**
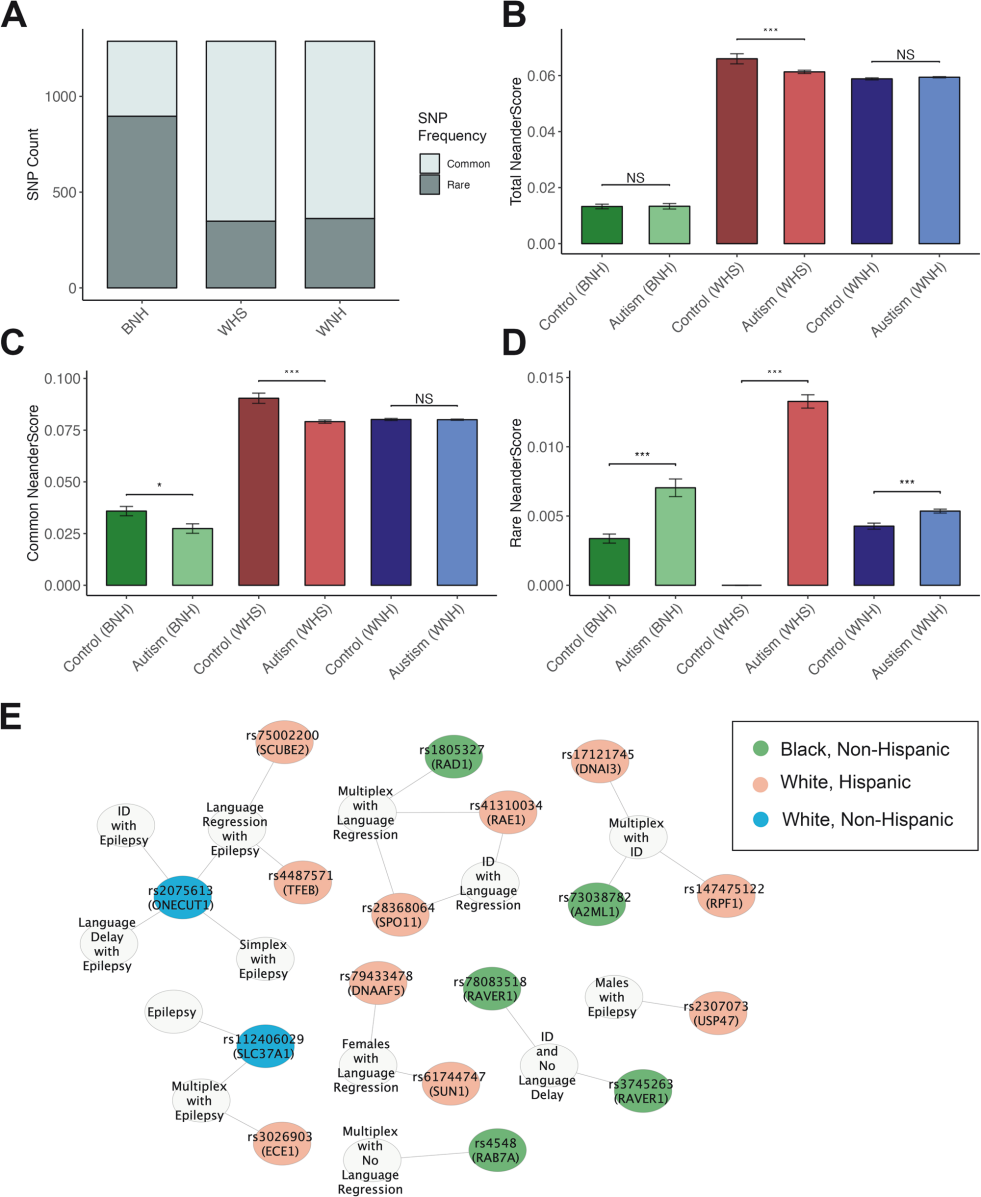
Group Comparison of SNP Frequencies and Quantitative Trait Loci (QTL) Network. **A** Rare and common SNP frequencies by ethnic control group. **B** Barplot with comparative Total NeanderScores between controls and autism broken down by ethnicity. No significant differences between autism and controls except in the white Hispanic (WHS) group. **C** Common NeanderScores across experimental and control groups by ethnicity. In general, autism groups—with the exception of white, non-Hispanics (WNH)—have lower common NeanderScores. **D** Rare NeanderScores of autism and controls by ethnicity. Note that all autism groups have significantly higher Rare NeanderScores compared to controls. (Due to small participant numbers, the control WHS group likely shows far lower Rare NeanderScores than they would with a larger N, resulting in an unusually dramatic deviation between controls and autism. Therefore, this particular result should be interpreted with caution.) **E** Cytoscape network illustrating clinical associations in autism (white nodes) with various brain-associated QTLs (colored nodes) that were either rare in autism or significantly enriched. SNPs are color-coded according to ethnic group. (Salmon = BNH, green = WHS, blue = WNH.).

Autism SNPs were categorized as “rare” (<1%) and “common” (≥1%), according to their frequency within the respective GTEx/1000G control datasets [27] (see Fig. 1A for rare-to-common SNP ratios by ethnic control group). Across all groups, homozygous ancestral (non-Neanderthal) SNPs were assigned a value of “0,” heterozygous Neanderthal SNPs were given a value of “1,” and homozygous Neanderthal alleles assigned the value of “2.” Raw NeanderScores were then calculated according to the average of these values per individual (divided by total possible SNPs, e.g., 1288 for Total NeanderScores) and larger group averages were subsequently broken down into the rare and common categories for additional analysis. The term, “NeanderScore,” is used here to refer to the average SNP enrichment in any given individual, with “Rare, “Common,” and “Total” NeanderScores referring to averages across groups of SNPs with varying frequencies. For instance, a given individual’s “Rare NeanderScore” within the white non-Hispanic group is calculated by tallying the total number of hits per person (0 = homozygous ancestral, 1 = heterozygous Neanderthal, 2 = homozygous Neanderthal) across the 348 rare SNPs and then averaged by dividing with the total number of possible hits (i.e., 348). This provides a percentage of enrichment, which, along with other individuals’ Rare NeanderScores, can then be used to determine group means and variance for comparison.

For enrichment analyses, all SNPs enriched by ≥ 5% in either the black non-Hispanic or white non-Hispanic autism groups relative to controls, or enriched by ≥ 10% in the white Hispanic autism group, were selected for additional investigation. Although these cutoffs are somewhat arbitrary, the white Hispanic cutoff was set higher relative to the other two ethnic groups due to greater deviation between white Hispanic autism and control data, which is likely due to poorer match of the control group and therefore we felt warranted a more conservative cutoff (see Fig. 1B). SNPs that achieved these cutoffs were statistically analyzed for enrichment relative to controls using two-proportion *Z* tests with Benjamini-Hochberg (BH) multitest correction. Finally, all SNPs that retained significance after correction but were not expressed in brain according to GTEx’s *Locus Browser (Variant-centric)*, which allows visualization of QTLs by tissue type, were also removed. This resulted in a final list of 6 brain-related QTLs in 5 host genes that were significantly enriched in the black non-Hispanic autism group, 18 SNPs in 15 host genes in the white Hispanic group, and 1 SNP in the white non-Hispanic group (25 in total). Gene consequences were derived from *dbSNP* [28]. Using NIH’s *LDexpress* tool, a list of host genes with SNPs in significant linkage disequilibrium in brain with our 25 SNPs of interest was also produced [29]. The standard settings were used, which included a window of 500k bp and *R*^*2*^ > 0.1, and all brain-related tissues were selected prior to search, such as “Brain – Cortex,” “Brain – Cerebellum,” etc. For each of the 6 SNPs enriched in black non-Hispanics, African Ancestry in Southwest USA (ASW) was used as a population background, as not all SNP pairings share LD on all genetic backgrounds. Meanwhile, both the MXL and PUR populations were used as a combined background for the 18 white Hispanic SNPs. And, finally, Northern Europeans from Utah (CEU) were used as background for white non-Hispanics. This resulted in a list of 43 host genes with additional brain-related QTLs of potential interest.

The original 25 SNPs were then analyzed for clinical associations available via the SPARK dataset. All other rare SNPs not significantly enriched in the autism groups were also assessed for clinical associations using *t*-tests/Mann-Whitney U tests and ANCOVAs, as they may still play important roles in comorbid clinical phenotypes despite a lack of relative enrichment compared to controls. Only SNPs that were brain-related QTLs were retained for the production of the genotype-phenotype Cytoscape network.

Finally, all rare SNPs enriched (not necessarily significantly) in our respective autism groups were analyzed for various functional enrichment patterns (e.g., Gene Ontology, KEGG Pathway, REAC, transcription factor binding sites) using the *g:Profiler* platform, which provides a list of significant enrichment terms as well as FDR-adjusted p-values [30].

### Statistics

For NeanderScores that followed a normal distribution, parametric statistics were used (i.e., *t-*test, ANOVA), with standard *post hoc* comparison when appropriate. Meanwhile, for datasets that were skewed (Shapiro-Wilk) or displayed unequal variances (Levene’s), nonparametric analyses such as Mann-Whitney U, Welch *t-*test, and Kruskal-Wallis were used, with Games-Howell *post hoc* comparison when appropriate. For binomial data (e.g., presence/absence), *Chi*-square and odds ratios were used. For covariates such as “Language Regression x Epilepsy,” ANCOVAs were used. Means and standard deviations for each analysis are available in the Supplementary Materials, as are all statistical analyses. Analyses were conducted and plots produced using R and Cytoscape, the former with the *ggplot2* package.

## Results

Within the present work, “NeanderScore” refers to an average of the Neanderthal DNA content within a given person’s genome, calculated based on the total number of Neanderthal-derived SNPs surveyed [7, 24]. NeanderScores were further divided into “Rare” and “Common” for additional analyses. All NeanderScores for the current study were calculated using only genic content and did not analyze intergenic regions.

Total NeanderScores differed significantly between ethnic groups as expected, with NeanderScores highest in white Hispanic people, followed by white non-Hispanics, and finally black non-Hispanics (*H* = 525.172, *p* = 2.939 × 10^−111^, *η*^2^ = 0.532] (Fig. 1B). However, according to *posthoc* analysis, Total NeanderScores did not differ between black non-Hispanic and white non-Hispanic experimental and control groups, although white Hispanic controls had significantly higher Total NeanderScores than their SPARK counterparts, which may be a reflection of small control numbers (*p* = 0.004) (Supplementary Materials - Table 1).

Rare SNPs occur, by definition, in less than 1% of the general population, while common in *≥*1% of the population [27]. Within each ethnic grouping, SNPs were broken down into these two categories (rare and common) according to their frequency within the GTEx or 1000G control groups and analyzed accordingly. Utilizing this approach, we investigated potential enrichment of common Neanderthal-derived SNPs in our autism groups compared to ethnically matched controls. Both the black non-Hispanic and white Hispanic autism groups exhibited significantly lower Common NeanderScores (i.e., had fewer common Neanderthal variants) (black non-Hispanic: *W* = 4617.500, *p* = 0.004; white Hispanic: *t*(88.520) = −4.355, *p* = 3.568 ×10^-5^). Meanwhile, autistic white non-Hispanics and their sibs did not significantly differ from the control group nor each other (*F*(2, 2879) = 2.225, *p* = 0.108) (Fig. 1C) (Supplementary Materials - Table 2).

In contrast to Total and Common NeanderScores, it was apparent there was a dramatic enrichment of rare SNPs in all SPARK groups compared to ethnically matched controls (black non-Hispanics: *H* = 1767.500, *p* = 5.308 × 10^−9^; white Hispanic: *X*^*2*^ = 281.994, *OR* = 7.624, 95% CI (4.809, 10.440), *p* = 3.511 × 10^−61^; white non-Hispanic *post hoc*: *t* = −3.997, *p*_*Tukey*_ = 1.940 × 10^−4^) (Fig. 1D). According to Standard *post hoc* comparisons, white non-Hispanic autistic people did not significantly differ from sibs (*p*_*tukey*_ = 0.746) and sibs did not significantly differ from controls (*p*_*Tukey*_ = 0.354) as their scores hovered midway between the other two groups (Supplementary Materials - Table 3). Unfortunately, sibling numbers were too low in the other two ethnic groups to enable similar comparisons. Lastly, sex was not a significant predictor of rare SNP enrichment within the various autism groups (*p* = 0.271–0.830), although males in general tended to have higher scores.

In order to further test the validity of the rare SNP categorization, we performed a randomization test on white non-Hispanic rare SNPs as this autism group displayed the smallest divergence from controls. The difference of the means of Rare NeanderScores between autism and controls was 1.086 × 10^−3^. After 10,000 randomized repetitions, the average difference of the means was 4.191 × 10^−6^, which is significantly less than the observed difference (*p* = 1.000 × 10^−4^), indicating that the original result was not obtained at random and lends further support to these results.

Following these analyses, we performed functional enrichment studies on rare SNPs enriched (though not necessarily significantly) in each autism group using the *g:Profiler* platform. Across all three ethnic groups, terms relating to “cytoskeleton” and/or “cell projection” were over-represented in the rare enriched genes (see Table 2). Within the white Hispanic rare genes, terms such as “nervous system development” and “neurogenesis” were also over-represented.

**Table 2:** Significant trends in functional enrichment of rare SNPs across the three ethnic groups according to *g:Profiler*.

Group	Category	Term name	Term ID	P(adj)
BNH (217 genes)	GO: biological process	Cytoskeleton organization	GO:0007010	3.959 × 10^−2^
	GO: cellular compartment	Plasma membrane bounded cell projection	GO:0120025	4.830 × 10^−3^
		Cytoskeleton	GO:0005856	1.794 × 10^−2^
		Axoneme	GO:0005930	1.794 × 10^−2^
	REAC	Signaling by cytosolic FGFR1 fusion mutants	REAC:R-HSA-1839117	4.935 × 10^−2^
WHS (199 genes)	GO: biological process	Anatomical structure development	GO:0048856	3.713 × 10^−8^
		Cell-cell adhesion	GO:0098609	7.508 × 10^−5^
		Nervous system development	GO:0007399	3.396 × 10^−4^
		Plasma membrane bounded cell projection organization	GO:0120036	1.984 × 10^−3^
		Neurogenesis	GO:0022008	1.809 × 10^−2^
	GO: cellular compartment	Cell projection	GO:0042995	3.126 × 10^−3^
		Cytoskeleton	GO0005856	1.542 × 10^−2^
WNH (171 genes)	GO: biological process	Homophilic cell adhesion via plasma membrane adhesion molecules	GO:0007156	9.939 × 10^−5^
		Anatomical structure development	GO:0048856	1.106 × 10^−3^
		Cell adhesion	GO:0007155	1.298 × 10^−3^
		Developmental process	GO:0032502	9.000 × 10^−3^
		Cell junction organization	GO:0034330	2.743 × 10^−2^
		Negative regulation of multicellular process	GO:0051241	4.845 × 10^−2^
	GO: cellular compartment	Cell projection	GO:0042995	5.384 × 10^−3^
		Membrane	GO:0016020	2.815 × 10^−2^
		Plasma membrane bounded cell projection	GO:0120025	2.817 × 10^−2^
		Cell junction	GO:0030054	4.430 × 10^−2^

*GO* Gene Ontology, *KEGG* Kyoto Encyclopedia of Genes and Genomes, *REAC* Reactome *TF* TRANSFAC.

We next investigated significant enrichment of specific SNPs across the different ethnic groups. A total of 6 SNPs in the black non-Hispanic autism group, 18 SNPs in the white Hispanic group, and 1 SNP in the white non-Hispanic group were significantly enriched relative to controls and, according to the GTEx’s *Variant-centric Locus Browser*, are brain-associated QTLs (see Table 3, Supplementary Materials - Table 4). Numbers of black non-Hispanic sibs were too small for analysis; however, white Hispanic sibs showed a similar enrichment as their affected siblings and did not significantly differ from each other with the exception of a single SNP (rs117034642), which was even higher in unaffected sibs (*Z* = −2.776, *BH adj. p* = 0.049). There was no enrichment difference between autistics and sibs regarding the single enriched SNP in the white non-Hispanic group (*Z* = 0.759, *p* = 0.447). These 25 SNPs were then fed into NIH’s *LDexpress* tool, which resulted in a further 43 host genes containing brain-related QTLs in significant LD with our original SNPs of interest. Interestingly, within the white Hispanic autism group, there was a notable enrichment, not only of heterozygous variants in the 18 SNPs overrepresented in this group, but of homozygous variants as well (heterozygous: *W* = 17,822.500, *p* = 2.146 × 10^−13^; homozygous: *W* = 13,761.000, *p* = 1.878 × 10^−4^). Regarding the single SNP enriched in white non-Hispanics, this same trend was not seen (*p* = 0.174). This analysis was not performed on the black non-Hispanic group as none of the 6 SNPs occurred in homozygous form in this group.

**Table 3:** List of Neanderthal-derived brain-related quantitative trait loci (QTL) enriched in different ethnicities in autism.

Locus	Rs ID	Frequency in controls	Host gene(s)	Consequence (dbSNP)	Host genes with SNPs in LD
**Black, Non-Hispanic (BNH)**					
10:132208002:G>A	rs79220014	Rare	*STK32C*	*STK32C* : Non Coding Transcript Variant	*DPYSL4* *JAKMIP3*
17:14076741:A>T	rs2230351	Rare	*COX10*	*COX10* : Missense Variant (T>S)	*CDRT15*
5:34908772:T>C	rs1805327	Rare	*RAD1*	*RAD1* : Missense Variant (E>G)*TTC23L* : Intron Variant	*RAI14*
11:12008896:C>G	rs28411401	Rare	*DKK3*	*DKK3* : Intron Variant	*MICAL2*
6:44304547:G>C	rs74950428	Rare	*AARS2; POLR1C*	*POLR1C* : Intron Variant*AARS2* : Intron Variant	NA
6:44304587:C>A	rs74964556	Rare	*AARS2; POLR1C*	*POLR1C* : Intron VariantAARS2 : Intron Variant	NA
**White, Hispanic (WHS)**					
12:47784123:C>T	rs7306788	Common	*HDAC7*	*HDAC7* : Synonymous Variant	*AMIGO2* *ENDOU* *PCED1B* *TMEM106C* *RAPGEF3* *SLC48A1*
15:74195628:A>T	rs971756	Common	*STRA6*	*STRA6* : Missense Variant (L>M)	*MPI* *SCAMP2* *SEMA7A* *UBL7*
15:74195505:G>A	rs971757	Common	*STRA6*	*STRA6* : Intron Variant	*MPI* *SCAMP2* *SEMA7A* *UBL7*
20:3191844:A>G	rs73075075	Common	*DDRGK1*	*DDRGK1* : Intron Variant	*DNAAF9* *ITPA* *SLC4A11*
17:78222788:G>A	rs17882271	Common	*BIRC5*	*BIRC5* : Intron Variant	*TMEM235*
3:8733903:C>T	rs1974763	Common	*CAV3*	*CAV3* : Synonymous Variant	NA
20:63564680:C>T	rs3810487	Common	*HELZ2*	*HELZ2* : Missense Variant (R>K)	*GMEB2* *LIME1* *ARFRP1* *RTEL1* *ZGPAT* *SLC2A4RG* *STMN3*
15:83942878:G>A	rs12901723	Common	*ADAMTSL3*	*ADAMTSL3* : Intron Variant	*ALPK3* *GOLGA6L4* *HOMER2* *NMB* *WDR73*
10:132208002:G>A	rs79220014	Common	*STK32C*	*STK32C* : Non Coding Transcript Variant	*DPYSL4* *JAKMIP3*
21:42575315:A>C	rs112406029	Common	*SLC37A1*	*SLC37A1* : Intron Variant*LOC101928212* : 2KB Upstream Variant	*PDE9A* *RSPH1* *SLC37A1*
11:11956026:T>C	rs2307073	Common	*USP47*	*USP47* : Synonymous Variant	*DKK3*
6:44147432:G>A	rs4714759	Common	*TMEM63B*	*TMEM63B* : Missense Variant (V>M)*POLR1C* : Intron Variant	*MRPL14*
6:44151939:A>G	rs3734697	Common	*TMEM63B*	*TMEM63B* : Synonymous Variant*POLR1C* : Intron Variant	*MRPL14*
6:44149927:C>T	rs4714762	Common	*TMEM63B*	*TMEM63B* : Synonymous Variant*POLR1C* : Intron Variant	*MRPL14*
6:41734881:C>A	rs4487571	Rare	*TFEB*	*TFEB* : Intron Variant*MIR10398* : 2KB Upstream Variant	NA
1:85124159:A>G	rs17121745	Common	*DNAI3*	*DNAI3* : Missense Variant (T>A)	NA
1:84483058:T>C	rs147475122	Common	*RPF1*	*RPF1* : Intron Variant	NA
19:7731701:T>G	rs117034642	Common	*CLEC4G*	*CLEC4G* : Synonymous Variant	*CD209* *EVI5L* *CLEC4M* *TRAPPC5*
**White, Non-Hispanic (WNH)**					
21:42575315:A>C	rs112406029	Common	*SLC37A1*	*SLC37A1* : Intron Variant *LOC10192821*2 : 2KB Upstream Variant	*CBS* *PDE9A* *RSPH1*

For each variant, functional consequences are listed according to available data on *dbSNP*, as well a listing of host genes containing brain-related QTLs that are in significant linkage disequilibrium (LD) with the SNPs of interest according to *LDexpress*.

Following these analyses, we investigated additional clinical associations connected with Rare NeanderScores. Initially, we looked at relationships between the different autism-related diagnoses denoted within the SPARK database. Rare NeanderScores significantly differed by diagnosis in the white non-Hispanic group [*F*(3, 2000) = 2.665, *p* = 0.046], although none of the diagnostic groups significantly differed from each other in Standard *post hoc* comparison (*p* = 0.156-0.996), making this result difficult to interpret. The other two ethnic groups did not significantly differ in this way (black non-Hispanic: *F*(3, 68) = 2.241, *p* = 0.091; white Hispanic: *F*(3, 308) = 1.915, *p* = 0.127). We then performed additional analyses investigating relationships between enriched SNPs that are brain-related QTLs and autism comorbidities. Only one SNP (rs112406029, *SLC37A1* host gene) in particular survived multitest correction in the white non-Hispanic group (*BH adj*. *p* = 0.012) and was significantly associated with epilepsy, occurring in 39% of that sample compared to 26% in non-epileptics and 22% in controls (Fig. 1E, Table 4). In fact, approximately 8% of white non-Hispanic autistic people with seizures were homozygous for this variant, compared to 1.5% in those without epilepsy (*X*^2^ = 17.842, *p* = 2.400 × 10^−5^). The rs112406029 enrichment was even more dramatic when family type (multiplex vs. simplex) was added as a covariate [*F*(1, 1905) = 11.055, *p* = 9.014 × 10^−4^]. Sixty-seven percent of multiplex individuals with epilepsy carried at least one copy of this variant, while 25% of those were homozygous (Table 4, Fig. 1E). Additional clinical associations turned up in the other ethnic groups as well. For instance, in the black non-Hispanic autism group, the rs1805327 missense variant in the *RAD1* host gene, which is enriched in this group in general, is over-represented in multiplex cases with language regression (*F*(1, 66) = 8.766, *p* = 0.004; *post hoc*: *p*_*Tukey*_ = 0.010–0.025). This is in contrast to an inverse relationship between overall Rare NeanderScore and language regression in autistic black non-Hispanics: individuals with higher Rare NeanderScores are significantly less likely to experience language regression (*W* = 348, *BH adj. p* = 0.012). In autistic white Hispanics, almost 80% of males with epilepsy carried the rs2307073 synonymous variant within the epilepsy-associated *USP47* host gene, compared to ~1/3rd of other autistic cases and 15% in the matched controls [*F*(1, 308) = 9.494, *p* = 0.002; *post hoc*: *p*_*Tukey*_ = 0.008-0.045) [31]. This particular SNP shares LD with additional SNPs within the *DKK3* host gene, which is also implicated in the black non-Hispanic group and is involved in regulation of canonical Wnt signaling [32]. (See Supplementary Materials -Table 5 for full statistical results).

**Table 4:** List of Neanderthal-derived brain-related quantitative trait loci (QTL) that share associations with various autism comorbidities/characteristics by ethnic group.

Group	Locus	Gene	rs ID	dbSNP	Interaction	Subgroup enrichment
WNH	21:42575315:A>C	*SLC37A1*	rs112406029	*SLC37A1* : Intron Variant *LOC101928212* : 2KB Upstream Variant	NA	Epilepsy
WNH	21:42575315:A>C	*SLC37A1*	rs112406029	*SLC37A1* : Intron Variant *LOC101928212* : 2KB Upstream Variant	Family type * epilepsy	Multiplex with epilepsy
WNH	15:52789024:C>A	*ONECUT1*	rs2075613	*ONECUT1* : Synonymous Variant *LOC105370824* : 2KB Upstream Variant	Family type * epilepsy	Simplex with epilepsy
WNH	15:52789024:C>A	*ONECUT1*	rs2075613	*ONECUT1* : Synonymous Variant *LOC105370824* : 2KB Upstream Variant	ID * epilepsy	ID with epilepsy
WNH	15:52789024:C>A	*ONECUT1*	rs2075613	*ONECUT1* : Synonymous Variant *LOC105370824* : 2KB Upstream Variant	Language delay * epilepsy	Language delay with epilepsy
WNH	15:52789024:C > A	*ONECUT1*	rs2075613	*ONECUT1* : Synonymous Variant *LOC105370824* : 2KB Upstream Variant	Language regression * epilepsy	Language regression with epilepsy
BNH	3:128806410:C>T	*RAB7A*	rs4548	*RAB7A* : Synonymous Variant	Family Type * language regression	Multiplex with no language regression
BNH	5:34908772:T>C	*RAD1*	rs1805327	*RAD1* : Missense Variant (E > G) *TTC23L* : Intron Variant	Family Type * language regression	Multiplex with language regression
BNH	12:8857630:A>G	*A2ML1*	rs73038782	*A2ML1* : Intron Variant	ID * family type	Multiplex with ID
BNH	19:10328622:A>G	*RAVER1*	rs78083518	*RAVER1* : Intron Variant	ID * language delay	ID and no language delay
BNH	19:10328825:C>T	*RAVER1*	rs3745263	*RAVER1* : Intron Variant	ID * language delay	ID and no language delay
WHS	20:57330052:G>A	*SPO11*	rs28368064	*SPO11* : Intron Variant *LOC105372687* : 2KB Upstream Variant	Family type * language regression	Multiplex with language regression
WHS	20:57374816:G>T	*RAE1*	rs41310034	*RAE1* : Intron Variant	Family type * language regression	Multiplex with language regression
WHS	1:21227230:G>T	*ECE1*	rs3026903	*ECE1* : Intron Variant	Family type * epilepsy	Multiplex with epilepsy
WHS	20:57330052:G>A	*SPO11*	rs28368064	*SPO11* : Intron Variant *LOC105372687* : 2KB Upstream Variant	ID * language regression	ID with language regression
WHS	20:57374816:G>T	*RAE1*	rs41310034	*RAE1* : Intron Variant	ID * language regression	ID with language regression
WHS	11:9021971:A>G	*SCUBE2*	rs75002200	*SCUBE2* : Intron Variant	Language regression * epilepsy	Language regression with epilepsy
WHS	6:41734881:C>A	*TFEB*	rs4487571	*TFEB* : Intron Variant *MIR10398* : 2KB Upstream Variant	Language regression * epilepsy	Language regression with epilepsy
WHS	7:770427:G>A	*DNAAF5*	rs79433478	*DNAAF5* : Intron Variant	Sex * language regression	Females with language regression
WHS	7:869411:G>A	*SUN1*	rs61744747	*SUN1* : Synonymous Variant *LOC124901568* : Intron Variant	Sex * language regression	Females with language regression

*BNH* black non-Hispanic, *WHS* white Hispanic, *WNH* white non-Hispanic.

We extended this analysis by investigating relationships between clinical phenotypes and rare SNPs by group—particularly those SNPs that are brain-related QTLs. While there were no single phenotype/SNP pairings that survived multitest correction, there were a number of comorbid phenotypes that exhibited significant associations with particular rare SNPs (Fig. 1E, Table 4). A notable example includes the rs20756613 SNP within the *ONECUT1* host gene in the white non-Hispanic group and its association with epilepsy and various other clinical features, such as intellectual disability (ID) and language delay. The rs4487571 variant in the *TFEB* host gene, which is a transcription factor that acts as a master regulator of a variety of metabolic and immune pathways, is enriched in white Hispanics with language regression and epilepsy. Finally, the rs73038782 variant in the *A2ML1* host gene, an inhibitor of various proteases and linked with some cases of Noonan syndrome, is enriched in black non-Hispanics from multiplex families without ID [33]. (See Supplementary Materials - Table 6 for full statistical results).

## Discussion

Neanderthals experienced a prolonged genetic bottleneck, leading to greater retention of nonsynonymous mutations within their dwindling population, suggesting some of these variants may still be represented in the human genome today [34]. Juric et al. [35] found that these weakly deleterious variants, though retained in Neanderthals as a result of small population size, have been under purifying selection once they entered the *H. sapiens* background with access to a larger population. In support, Wei et al. [36] reported Neanderthal variants are significantly depleted in the modern genome relative to alleles matched for frequency and linkage disequilibrium.

Here we report an enrichment of a subset of rare and common Neanderthal-derived polymorphisms in autism across three major ethnic groups (black non-Hispanic, white Hispanic, and white non-Hispanic). The low frequency of some of these SNPs, along with their clinical associations, suggests they are weakly deleterious and under continued purifying selection. These trends are not, however, accompanied by a general enrichment in overall Neanderthal content in the SPARK groups, which suggests that not all Neanderthal-derived DNA is equally implicated in susceptibility.

### Neanderthal-derived variants in other neurodevelopmental conditions

Previous studies have investigated the potential roles of Neanderthal DNA in susceptibility to other neurodevelopmental conditions. For instance, Gregory et al. [37] reported that lower NeanderScores are associated with schizophrenia and, in particular, with the positive symptoms often present in the condition, suggesting *Homo sapiens*-specific variants, rather than Neanderthal, may be playing a role in susceptibility to the condition. We would point out, however, that these findings do not necessarily disagree with the current study. For instance, the present autism samples are not necessarily enriched in all frequency types of Neanderthal variants studied. While white Hispanic autistic people have a dramatic enrichment of rare variants, they have lower total NeanderScores compared to ethnically matched controls. A similar trend is seen in this group and black non-Hispanics when calculating common NeanderScores, i.e., variants that occur in ≥1% in controls. We are, therefore, proposing that a select subset of Neanderthal-derived variants are playing roles in autism susceptibility and other comorbid features, such as ID, epilepsy, and language regression. It would be interesting to determine whether a subset of Neanderthal-derived SNPs might not be playing similar roles in schizophrenia susceptibility, despite lower overall NeanderScores.

Along a similar line of research, Srinivasan et al. [12] identified lower rates of Neanderthal Selective Sweeps (Neanderthal variant-enriched regions) in portions of the genome that are over-represented by brain-related genes implicated in schizophrenia. However, it has previously been recognized that brain-related genes, including major effect genes implicated in autism, are tightly conserved and generally mutation intolerant as a result of dosage sensitivity [38]. A similar immutability is seen in schizophrenia-related genes [39]. Therefore, one would expect genomic introgression to be underrepresented in such regions following events such as hybridization. However, even small numbers of key variants in such genes, as a result of their relative intolerance, may nevertheless be keenly felt. In addition, it is vital to understand the contexts under which *Homo sapiens-*specific variation occurred. Hybridization in non-human species is recognized as a destabilizing event with the potential to promote compensatory adaptation in other regions of the genome and, ultimately, speciation in some lineages [40]. Therefore, there is an additional possibility that *Homo sapiens*-specific variants that evolved after Neanderthal introgression may nevertheless be influenced by these earlier events.

### Neanderthal DNA, Social Cognition, & Visual Processing

The roles that some Neanderthal-derived variants play in connectivity within the intraparietal sulcus and their concomitant influence on social abilities and visual processing have implications for some of the clinical features of autism. Given the results of the current study, some may ask whether features of autism are reminiscent of behaviors seen in Neanderthal people. Of relevance, recent evidence from numerous individuals from the Altai Mountains of southern Siberia has shown that their genomes held long segments of homozygosity, indicative of small breeding pools [41]. From these and other data, the authors make inferences about Neanderthals’ social organization, reinforcing previous notions that they likely lived in small communities (reviewed in [41]). Previous studies have found that social group size of a primate species is strongly predictive of overall neocortical size, suggesting links between group size and cognitive function [42, 43]. While Neanderthals appear to have maintained more regional patterns of social interaction, early European AMH seem to have engaged in broader, better-integrated social networks [44]. An interesting topic for future research is whether Neanderthal-derived sociobehavioral tendencies may be reflected in some people on the autism spectrum (a topic colloquially known as the “Neanderthal Theory of Autism”) [45].

Conversely, individuals on the autism spectrum often exhibit strengths in visuospatial processing [17, 19]. While an extensive array of tool-making techniques is associated with AMH in the Upper Paleolithic, it should be noted that the Levallois technique, which is most associated with Neanderthal societies in the Middle Paleolithic, is thought to require more skill and training than those necessary to produce the later Upper Paleolithic blades, indicating that Neanderthals were indeed capable of exceptional craftsmanship [46]. Recent research has also revealed additional decorative artistry in Neanderthal societies, with evidence of the use of flight feathers culled from birds of prey for probable personal adornment, as well as early examples of cave art [47, 48]. Relevant to the fields of paleoanthropology and paleoarcheology, there are numerous examples of autistic savants with exceptional visuospatial abilities (e.g., artistic ability) who nevertheless have significant challenges with verbal communication, suggesting that the use of visual symbolism in ancient humans is not necessarily an adequate proxy for presumed language ability as these are modular functions.

Pertinent to the topic of autistic abilities and savantism, although most studies on autism genomics focus on the deleterious nature of variants, there is the possibility some of these autism-associated Neanderthal SNPs have been under weak positive selection. In support, recent studies have identified genetic variants implicated in both autism and high intelligence [49]. Meanwhile, autistic people often perform better on tests of fluid intelligence than neurotypicals [50]. The variable penetrance of these Neanderthal variants for autism, as evidenced by similar patterns of enrichment in unaffected siblings, as well as enrichment of some common variants, suggest a means for their retention. In further support, studies on the cognitive ability of unaffected siblings show sibs tend to have higher performance IQ scores relative to verbal IQ – a pattern very similar to affected siblings and different from neurotypicals [51]. In addition, families of students studying disciplines like math, physics, and engineering are more likely to have autistic family members than students studying the humanities, suggesting an extended cognitive phenotype exists [52].

Of potential relevance to this topic, paleoarcheologists have identified an “Upper Paleolithic Revolution” beginning around 50 kya, which was a change in the ways AMH made tools, traveled and engaged in trade, used and produced artistic materials, and structured their living habitats, such as dividing the home into food preparation, discard, cooking, and sleeping areas [53]. Interestingly, this time period roughly coincides with hybridization between *H. sapiens* and Neanderthals, suggesting hybridization may have been a stimulus for cognitive change, one which may continue to influence intellectual ability and susceptibility to neurodevelopmental conditions in modern humans. This has been hypothesized before, although from a sociocultural rather than a genetics perspective [54]. Further work is needed to address these possibilities.

### Limitations

Despite strong clinical associations reported here, analyzing large numbers of SNPs gives the viewer only a gross perspective of potential roles Neanderthal-derived SNPs may be playing in autism susceptibility. While a subset of variants may be playing complex roles in susceptibility, there is much noise surrounding this genic signal and the current work would benefit in future from greater magnification of networks of SNPs potentially driving these results.

There are additional limitations we believe may have restricted or influenced the current results. The first concerns limitations within some of the clinical data made available in the SPARK database, most notably the lack of ASD levels (1-3), which subsequently limited our ability to address potential enrichment differences and SNP associations by severity level (although data on comorbid ID were made available, as were pre-DSM-5 diagnoses). Another limitation concerns issues surrounding personalized medicine and population genetics: while there is an ever-growing array of population-based genomic material made available for research, the genetic backgrounds of subpopulations can vary significantly from different collection sites even across identical self-reported ethnic labels. In addition, despite ethnic labels, humans are often significantly admixed, which can influence these types of studies. This emphasizes the importance not only of continued efforts to identify ethnic backgrounds genetically and to rely less on self-reported identification for the purposes of genetics studies, but to also stress the importance of collecting non-clinical control data from the same clinical sites as the experimental groups, which may help reduce background variability within these self-reported labels. Related to this, it was challenging to acquire an appropriate ethnic control match for white Hispanics within the SPARK dataset, which we suspect has likely led to the insertion of noise within the results. In future iterations of this research, we will be utilizing admixture analysis rather than relying solely on self-reported labels for group assignment.

The final limitation concerns previous research on the Neanderthal genome. While Neanderthal introgression has indeed been confirmed across multiple studies through a variety of algorithms, it has nevertheless been challenging to differentiate introgression from incomplete lineage sorting (ILS), the latter due to common ancestry and not hybridization. Unfortunately, methods parsing introgression from ILS tend to have poor specificity at the SNP level [55]. Therefore, it is possible that some of the SNPs reported in the present work may be a result of ILS rather than hybridization and may instead be shared by a deeper human ancestry, which holds interesting possibilities regarding human brain evolution itself. Future studies utilizing new algorithms will undoubtedly continue to improve on the available data and provide better clarity regarding these questions.

## Conclusion

This is the first study to provide strong evidence for the active role of a subset of rare, as well as some common, Neanderthal-derived alleles in autism susceptibility in multiple major American populations. We hope this research will lead to further investigation into the ongoing influences of ancient hybridization between *H. sapiens* and Neanderthals in brain development, human intelligence, and overall human health, as well as spur work into additional clinical resources for this complex population.

## Supplementary information

Supplementary Materials

## Footnotes

1 Within the current manuscript, the authors have elected to use identity-first language in accordance with current research on preferences of autism stakeholders [19].
